# Real-world treatment patterns and adverse events in metastatic renal cell carcinoma from a large US claims database

**DOI:** 10.1186/s12885-019-5716-z

**Published:** 2019-06-07

**Authors:** Sumanta Pal, Jun Gong, Shivani K. Mhatre, Shih-Wen Lin, Andy Surinach, Sarika Ogale, Rini Vohra, Herschel Wallen, Daniel George

**Affiliations:** 1 Department of Medical Oncology and Experimental Therapeutics, City of Hope National Medical Center, 1500 East Duarte Road, Duarte, CA 91010 USA; 2 Department of Hematology/Oncology, City of Hope National Medical Center, 1500 East Duarte Road, Duarte, CA 91010 USA; 3 Real World Data Science (Oncology), Genentech, Inc, 1 DNA Way, MS 352B, South San Francisco, CA 94080 USA; 4 Genesis Research, 5 Marine View Plaza, Hoboken, NJ 07030 USA; 5 US Medical Affairs, Genentech, Inc, 1 DNA Way, MS 352B, South San Francisco, CA 94080 USA; 6 School of Pharmacy, West Virginia University, P.O. Box 9500, Morgantown, WV 26506 USA; 7 Oncology and Hematology Care Clinic, Providence Cancer Center, 4805 NE Glisan Street, Suite 6N40, Portland, OR 97213 USA; 8 Department of Medicine, Medical Oncology, Duke University School of Medicine, Box 103861, Durham, NC 27710 USA

**Keywords:** Treatment patterns, Adverse events, Renal cell carcinoma, Targeted therapy, Administrative claims

## Abstract

**Background:**

Vascular endothelial growth factor (VEGF), tyrosine kinase (TK) and mechanistic target of rapamycin kinase (mTOR) inhibitors are common first-line (1 L) treatments for metastatic renal cell carcinoma (mRCC). Despite treatment availability, the 5-year survival rate in patients diagnosed at the metastatic stage is only ≈ 10%. To gain contemporary insights into RCC treatment trends that may inform clinical, scientific and payer considerations, treatment patterns and adverse events (AEs) associated with 1 L therapy were examined in a retrospective, longitudinal, population-based, observational study of patients with mRCC.

**Methods:**

US administrative claims data (Truven Health MarketScan Commercial Databases) were used to assess trends in 1 L treatment initiation in mRCC (2006–2015) and characterize patterns of individual 1 L treatments, baseline characteristics, comorbidities and treatment-related AEs from 2011 through 2015. Outcomes were evaluated by drug class and route of administration.

**Results:**

Ten-year trend analysis (*n* = 4270) showed that TK/VEGF-directed therapy rapidly became more common than mTOR-directed therapy, and oral treatments were favored over intravenous (IV) treatments. Overall, 1992 eligible patients initiated 1 L treatment for mRCC from 2011 through 2015: 1752 (88%) received TK/VEGF-directed agents and 233 (12%) received mTOR-directed agents; 1674 (84%) received oral treatments, and 318 (16%) received IV treatments. The most common 1 L treatment was sunitinib (*n* = 849), followed by pazopanib (*n* = 631), temsirolimus (*n* = 157) and bevacizumab (*n* = 154). Patient characteristics and comorbidities, including age, diabetes and congestive heart failure, were independent predictors of 1 L mRCC treatment choice. The three most common potentially 1 L treatment–related AEs were nausea/vomiting (128.2 per 100 patient-years [PY]), hypertension (69 per 100 PY) and renal insufficiency (44.6 per 100 PY). A wide variety of agents were used as second-line (2 L) therapy. Substantial latency of onset was observed for several potentially treatment-related toxicities in patients treated with TK/VEGF- or mTOR-directed agents.

**Conclusions:**

In the US, 1 L TK/VEGF inhibitor uptake in recent years appears largely in line with national approvals and guidelines, with varied 2 L agent use. Although retrospective evaluation of claims data cannot assess underlying causality, insights from these real-world RCC treatment and AE patterns will be useful in informing medical and payer decisions.

**Electronic supplementary material:**

The online version of this article (10.1186/s12885-019-5716-z) contains supplementary material, which is available to authorized users.

## Background

Kidney cancer is the eighth most common cancer in the United States [1]. Renal cell carcinoma (RCC) is the most common type of adult kidney cancer, making up about 90% of diagnoses [2]. Approximately 65% of patients with newly diagnosed RCC have localized disease at the time of diagnosis, while 25 to 30% have advanced or metastatic disease [1, 3]; approximately 20 to 40% of patients with localized RCC will progress to metastatic RCC (mRCC) [4]. The prognosis associated with mRCC is poor, with a 5-year relative survival rate of approximately 8 to 12% in patients initially diagnosed with distant metastatic disease [1, 5]. Progression-free survival (PFS) has been shown to predict overall survival (OS) in patients with mRCC [6]; therefore, first-line (1 L) treatment for mRCC is crucial to improving outcomes.

In the last decade, several tyrosine kinase (TK) inhibitors, vascular endothelial growth factor (VEGF) inhibitors (collectively, TK/VEGF inhibitors) and mechanistic target of rapamycin (mTOR) inhibitors have been approved in the United States for 1 L use in patients with mRCC. These agents have notably improved efficacy [4] compared with interferon (IFN)-α and interleukin 2 (IL-2), the previous standards of care [7, 8]. Sunitinib and pazopanib, both multitargeted TK inhibitors that also target VEGF, have been shown to improve objective response rate (ORR) and PFS compared with either IFN-α or placebo, respectively [9, 10], and pazopanib was also found to be non-inferior to sunitinib with respect to PFS, with similar OS observed [11]. For 1 L treatment of clear cell RCC, current National Comprehensive Cancer Network (NCCN) guidelines recommend several treatment options with distinct mechanisms of action, including sunitinib or pazopanib as category 1 options [8]. Since the introduction of these agents, however, data on their use in routine clinical practice have been limited [12–14] and have tended to focus on specific patient subgroups or selective treatments. With the changing landscape in the 1 L treatment of mRCC, an understanding of real-world treatment use, sequencing and associated adverse events (AEs) of historic but widely used and recommended current standard of care treatments is required [15, 16] and can provide practitioner, researcher and payer insights into the care and benefit:risk profiles of mRCC treatment.

## Methods

### Study objectives

The aims of this retrospective, longitudinal, population-based, observational analysis were to (1) assess 10-year trends in 1 L mRCC treatment initiation by drug class and route of administration (from 2006 through 2015) and (2) characterize the patterns of individual 1 L treatments and baseline characteristics and comorbidities of treated patients (from 2011 through 2015). Additionally, in exploratory analyses, clinical factors and potentially treatment-related AEs associated with 1 L treatments during the latter period were assessed by drug class and route of administration.

### Study design and databases

**Fig. 1:**
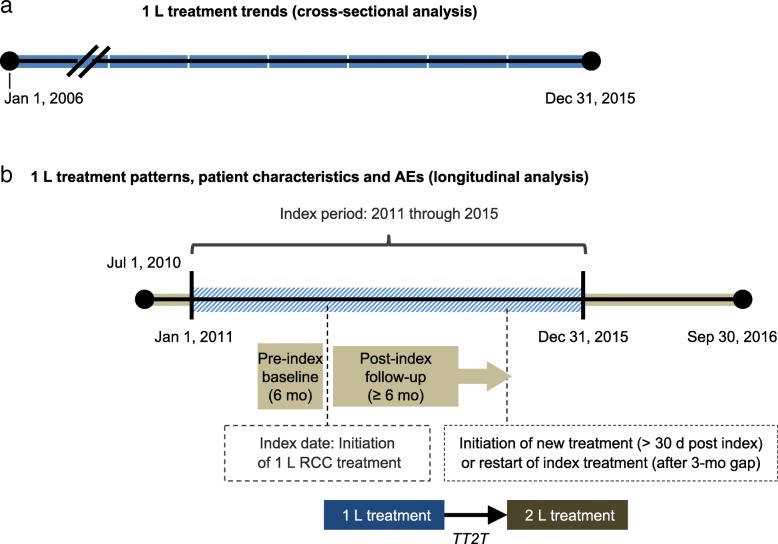
Study design. **a** Trend analysis. **b** Treatment patterns, patient characteristics and AE analysis. The study periods are indicated as durations between closed circles. The index date (date of 1 L treatment initiation for mRCC) could occur during the index period. Patients were followed up for a minimum of 6 months until the end of continuous enrollment. The end of 1 L treatment was defined as the last 1 L treatment claim date plus (1) the number of days of supply of the oral treatments (last claim) or (2) the labeled cycle length for the IV treatments. Time to 2 L treatment (TT2T) was defined as the initiation of a new 1 L therapy regimen > 30 days following the index date or restart of 1 L index treatment following a > 3-month gap

US administrative claims data from the Truven Health MarketScan Commercial Claims and Encounters (Commercial) and Medicare Supplemental and Coordination of Benefits (Medicare) Databases were used in this study. Cross-sectional data were used for the trend analysis, and longitudinal data were used for all other study objectives (Fig. 1). The MarketScan Research Databases make up the largest private-sector healthcare database in the United States and include information from employer-sponsored plans that provide health benefits to over 15 million people annually, including employees, their spouses and dependents, approximately 10% of whom are aged ≥ 65 years. All data on treatment utilization and medical conditions were derived from records of prescription claims for treatment and medical claims for conditions, respectively. This study used Truven MarketScan retrospective administrative claims data. Data were de-identified and comply with the Health Insurance Portability and Accountability Act and the 1964 Helsinki Declaration and its later amendments or comparable ethical standards. Thus, Institutional Review Board approval was not required, and formal informed consent was not obtained.

### Patient populations and treatment definitions

For the 10-year trend analysis, patients aged ≥ 18 years with mRCC who initiated 1 L treatment between January 1, 2006, and December 31, 2015, were included (Fig. 1a). For analyses of treatment patterns, patient characteristics and AEs, patients aged ≥ 18 years with mRCC who initiated 1 L treatment between January 1, 2011, and December 31, 2015 (total study period July 1, 2010 to September 30, 2016), were included (Fig. 1b). The index date was defined as the date of 1 L treatment initiation for mRCC, and patients were followed for a minimum of 6 months until the end of continuous enrollment. Eligibility criteria are shown in Table 1. Based on similar mechanisms of action, agents were grouped as TK (sunitinib, sorafenib, pazopanib and axitinib)/VEGF inhibitors (bevacizumab ± IFN-α) or mTOR-directed therapy (temsirolimus and everolimus). These agents were classified by route of administration as oral (sunitinib, sorafenib, pazopanib, axitinib and everolimus) or intravenous (IV; bevacizumab ± IFN-α, temsirolimus and IL-2).

**Table 1:** Eligibility criteria (longitudinal analysis)

	Patients, n
Inclusion criteria	
≥ 2 claims with *ICD-9* code 189.0 or *ICD-10* code C64.xx for malignant neoplasm of the kidney on separate dates (≥ 30 days apart) during the index date period	77,565
First claim (ever) for a first-line mRCC agent (sunitinib, pazopanib, bevacizumab ± IFN-α^a^, temsirolimus, everolimus, sorafenib, axitinib, interleukin 2) occurs during the index date period, within 30 days before the first mRCC claim and up to the end of the rolling index period (patients with two different agents within 5 days of each other were excluded)	5813
Aged ≥ 18 years at index date	5788
Continuous enrollment eligibility 6 months pre-index and 6 months post-index dates with no gap	2565
Total no. of patients after applying all the inclusion criteria	2565
Exclusion criteria	
Evidence of TCC (≥ 1 claim) from 30 days pre-index date through the entire follow-up period. TCC was identified by receipt of agent indicated for TCC	2426
≥ 2 claims for 1 primary cancer from 6 months pre-index date through the index date (except for sites to which primary RCC commonly metastasizes, such as lung, bone, brain and liver)	1992

^a^ For the combination treatment with bevacizumab + IFN-α, the index date is the first occurring claim of either agent. The other drug claim must have occurred within a period of 30 days after the first agent claim to qualify as combination treatment. *TCC* transitional cell (urothelial) carcinoma

### Measures

Treatment duration was defined as the number of days from the index date to either (1) the end of the treatment regimen (with a ≤ 3-month gap permitted) or (2) a switch to a new treatment after 30 days following the index date. End of 1 L treatment was calculated as the last 1 L treatment claim date plus (1) the number of days’ supply of oral treatments (last claim) or (2) the labeled cycle length for the IV treatments. In the event of a change to a new treatment within 30 days of the initial index date, followed by a subsequent refill of the second drug, the second drug was considered the 1 L index drug and initiation of the second drug was then defined as the 1 L index date. Time to 2 L treatment (TT2T) was defined as the initiation of a new treatment more than 30 days following the index date or restart of 1 L index treatment following a > 3-month gap. Baseline comorbidities were identified and incorporated into the modified Deyo-Charlson Comorbidity Index (DCCI) score (including non-cancer comorbidities), whereby a higher score reflects a high comorbidity burden [17, 18]. Secondary metastases were identified using ICD-9/ICD-10-CM codes and categorized as lung (197.0–197.3, C78.0-C78.3), liver (197.7, C78.7), brain (198.3, 198.4, C79.3, C79.49), bone (198.5, C79.5) and other sites.

Potentially treatment-related AEs were identified using corresponding *International Classification of Diseases (ICD), Ninth Revision* and *ICD, Tenth Revision, Clinical Modification* codes in the administrative claims data that occurred during the treatment duration up to 30 days after the last 1 L drug claim. Such codes are likely associated with medical attention, though administrative claims databases are unable to provide definitive causality. The AEs evaluated in this study were chosen from searching the product labels for each of the drugs described, and only those that were reported to be grade 3 or 4 severity and occurring with a > 5% incidence were included [19–25]. Additional AEs, such as diarrhea, fatigue/asthenia and hand-foot syndrome, which are commonly associated with checkpoint inhibitors, were also included in order to provide historical estimates for the targeted therapies (Additional file 1: Table S1).

### Statistical analysis

All analyses were assessed as a function of the 1 L index treatment, including drug class and route of administration. Categorical variables were reported as counts and percentages, and continuous variables were reported as means with standard deviations or medians with interquartile ranges (Q1–Q3). For the 10-year trend analysis, the proportion of patients receiving the index treatment by drug class and route of administration each year was determined.

Treatment patterns were evaluated as the number and percentage of patients receiving each 1 L treatment and switching to the corresponding second-line (2 L) treatment, the duration of 1 L treatment and TT2T. Kaplan-Meier methods (median, 95% CI) were used for the duration of 1 L treatment and TT2T. The duration of 1 L treatment was evaluated to measure the difference in time from the index date to the end of 1 L treatment, with an event defined as discontinuation of 1 L treatment and switch to a 2 L treatment or discontinuation of 1 L treatment > 3 months prior to the enrollment eligibility end date (for patients who did not switch to a 2 L therapy). Patients who discontinued 1 L treatment within 3 months of the enrollment eligibility end date were censored at the enrollment eligibility end date. For TT2T evaluation, an event was defined as a switch to a 2 L treatment. Patients who did not switch were censored at the end of 1 L treatment or the end of continuous enrollment, whichever was earlier.

Differences in baseline patient characteristics by 1 L treatment class (TK/VEGF inhibitor vs mTOR inhibitor) and route of administration (oral vs IV) were compared using a univariate *t* test and χ^2^ test (or Fisher exact test when appropriate) for continuous and categorical variables, respectively. Variables found to be statistically significant (*P* < 0.2) in the univariate analyses were used in the multivariate logistic regression to model the odds of choosing the drug class or route of administration. Irrespective of the *P* value in the univariate analysis, age, sex and index year were covariates in the multivariate analysis.

AEs were reported as incidence rate per 100 patient-years (IR per 100 PY) in the total population and in subpopulations by drug class and route of administration. IR was calculated as the number of patients with at least one AE after the index date, divided by the total PY at risk. If a patient did not have an AE, his/her person-time was counted up to 30 days after the end of 1 L treatment or the end of eligibility, whichever occurred first. A washout period of 30 days prior to the index date was applied to exclude patients with pre-existing AEs of interest when identifying incidence of non-chronic AEs. For chronic conditions, such as hypertension, hypotension, hepatitis, thyroid disorders, renal insufficiency, adrenal insufficiency and myasthenia gravis, a 365-day washout period was applied to identify true incident events.

## Results

### Ten-year trends in 1 L treatment initiation for mRCC: cross-sectional analysis

**Fig. 2:**
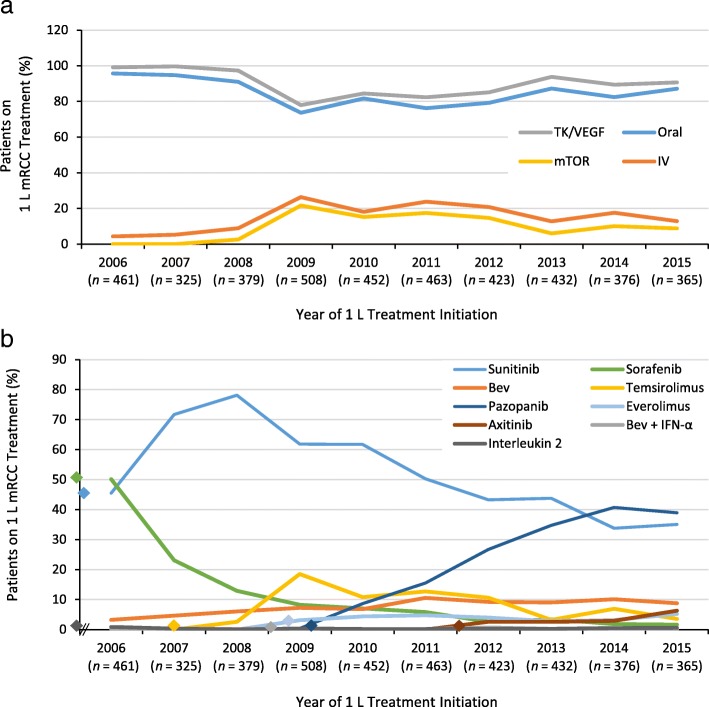
Trends in 1 L treatment for mRCC by drug class and route of administration (2006–2015). **a** Trends by drug class and route of administration. TK/VEGF inhibitors included sunitinib, pazopanib, sorafenib, axitinib and bevacizumab ± IFN-α. mTOR treatments included temsirolimus and everolimus. Orally administered agents included sunitinib, pazopanib, sorafenib, axitinib and everolimus; IV treatments included bevacizumab ± IFN-α, temsirolimus and interleukin 2. For the oral (blue line) vs IV (orange line) comparison, the percentage of each group sums to 100% for each year. For the TK/VEGF (grey line) vs mTOR (yellow line) comparison, the percentage of each group may sum to < 100% because interleukin 2 was not included (accounts for < 1% of patients in each year). **b** Trends by individual treatment. Trends are plotted as a single value for the entire year, and approximate timings for US FDA approvals are indicated by diamond symbols in part b (pre-2006 approvals are shown adjacent to the y-axis). Approvals plotted include 1 L sunitinib (January 26, 2006), 1 L pazopanib (October 19, 2009), 2 L sorafenib (December 20, 2005), 2 L axitinib (January 27, 2012), 1 L bevacizumab ± IFN-α (July 31, 2009), 1 L temsirolimus (May 30, 2007), 2 L everolimus (March 30, 2009) and 1 L interleukin 2 (May 5, 1992). In each given year, the percentage of patients receiving each indicated agent sums to 100%

Between 325 and 508 patients were evaluated each year from 2006 through 2015 (Fig. 2). During the 10-year study period, TK/VEGF-directed treatments were much more commonly initiated than were mTOR-directed or IL-2 treatments. Initiation of TK/VEGF-directed agents ranged from 91 to 95.7% from 2006 through 2009, dropped to 70.5% in 2009 and ranged from 71.5 to 84.5% between 2009 and 2015 (Fig. 2a). Starting in 2009 and extending through 2015, a downward trend for sunitinib (61.8–35.1%) and an upward trend for pazopanib (0.4–38.9%) were observed (Fig. 2b). Initiation of VEGF-directed therapy, consisting primarily of bevacizumab monotherapy, ranged from 3.3 to 10.1% (2006–2015), without any distinct trend. mTOR inhibitor use, predominantly temsirolimus, increased rapidly from 2.6% in 2008 to 21.7% in 2009 followed by a downward trend to 8.8% in 2015. During the study period, oral treatments were also much more common that IV treatments, although initiation of oral treatments dropped from 95.7 to 73.6% and that of IV treatments increased from 4.3 to 26.4% between 2006 and 2009. After 2009, oral initiation ranged from 76.2 to 87.3% and IV initiation ranged from 12.7 to 23.8% (Fig. 2a).

### Baseline clinical and demographic characteristics and 1 L treatment patterns: longitudinal analysis

Overall, 1992 patients initiated 1 L treatment for mRCC from 2011 through 2015 and were eligible for the longitudinal analysis (Table 1). The cohort had a median (Q1–Q3) follow-up of 15.6 (10.2–25.9) months. The median age was 62 years, and the majority of patients were male (70%) and largely from urban areas (82%). The mean modified DCCI score was 0.7, and 63% of patients had no reported comorbidities. The most common comorbidities (in > 10% of patients) were diabetes (27%), chronic kidney disease (20%), liver disease (18%) and chronic obstructive pulmonary disease (12.6%). Characteristics of 1 L treatment subgroups are shown in Table 2.

**Table 2:** Key baseline characteristics of patients initiating 1 L RCC treatment (2011–2015): drug class and administration route

Characteristic	TK/VEGF inhibitor (*n* = 1752)		mTOR inhibitor (*n* = 233)		Oral (*n* = 1674)		IV (*n* = 318)	
	n	%	n	%	n	%	n	%
Age at diagnosis, median (Q1–Q3), years	62 (56–70)		62 (56–71)		61 (56–69)		64 (57–76)	
Male	1220	69.6	166	71.2	1176	70.3	215	67.6
Employment status								
Active	568	32.4	69	29.6	563	33.6	79	24.8
Retiree	652	37.2	78	33.5	617	36.9	113	35.5
Long-term disability	11	0.6	1	0.4	8	0.5	4	1.3
Other/unknown	521	29.7	85	36.5	486	29.0	122	38.4
Region								
Northeast	327	18.7	33	14.2	303	18.1	57	17.9
North Central	467	26.7	52	22.3	451	26.9	68	21.4
South	645	36.8	104	44.6	622	37.2	131	41.2
West	296	16.9	40	17.2	280	16.7	59	18.6
Unknown	17	1.0	4	1.7	18	1.1	3	0.9
Metropolitan statistical area								
Urban	1424	81.3	195	83.7	1359	81.2	267	84.0
Rural	328	18.7	38	16.3	315	18.8	51	16.0
Insurance plan type								
Comprehensive	313	17.9	45	19.3	288	17.2	70	22.0
HMO	204	11.6	22	9.4	189	11.3	38	12.0
POS	130	7.4	17	7.3	120	7.2	28	8.8
PPO	926	52.9	125	53.7	896	53.5	158	49.7
Other	179	10.2	24	10.3	181	10.8	24	7.6
Insurance type								
Commercial	1120	63.9	142	70.0	1100	65.7	169	53.1
Medicare	632	36.1	91	39.1	574	34.3	149	46.9
Index year								
2011	350	20.0	78	33.5	341	20.4	88	27. 7
2012	350	20.0	62	26.6	333	19.9	80	25.2
2013	392	22.4	25	10.7	373	22.3	45	14.2
2014	327	18.7	38	16.3	309	18.5	58	18.2
2015	333	19.0	30	12.9	318	19.0	47	14.8
Mean DCCI score (Q1–Q3)	0.70 (0–1)		0.76 (0–1)		0.66 (0–1)		0.93 (0–1)	
Comorbidities								
Diabetes	484	27.6	48	20.6	442	26.4	90	28.3
CKD	346	19.8	54	23.2	317	18.9	84	26.4
Liver disease	307	17.5	40	17.2	306	18.3	45	14.2
COPD	217	12.4	33	14.2	212	12.7	39	12.3
CHF	113	6.5	37	15.9	111	6.6	40	12.6
Secondary metastatic sites								
Lung	798	45.6	101	43.4	804	48.0	99	31.1
Liver	181	10.3	39	16.7	186	11.1	34	10.7
Brain	161	9.2	25	10.7	162	9.7	26	8.2
Bone	469	26.8	85	36.5	479	28.6	76	23.9
Others	696	39.7	100	42.9	704	42.1	97	30.5
Nephrectomy prior to treatment								
Any nephrectomy	395	22.6	47	20.2	390	23.3	53	16.7
Cytoreductive nephrectomy	380	21.7	41	17.6	377	22.5	45	14.2

Data are n and % unless otherwise indicated. *CHF* congestive heart failure, *CKD* chronic kidney disease, *COPD* chronic obstructive pulmonary disease, *DCCI* Deyo-Charlson Comorbidity Index, *HMO* health maintenance organization, *POS* point-of-service, *PPO* preferred provider organization, *Q* quartile

Treatment patterns are included in Table 3. A total of 1752 patients (88%) received TK/VEGF inhibitor treatment, 233 (12%) received mTOR inhibitor treatment, 1674 (84%) received oral treatment and 318 (16%) received IV treatment. The most common 1 L treatment was sunitinib (*n* = 849), followed by pazopanib (*n* = 631), temsirolimus (*n* = 157) and bevacizumab (*n* = 149). A total of 154 patients were treated with bevacizumab, but only 3% (*n* = 5) received bevacizumab in combination with IFN-α. Baseline characteristics by agent are shown in Additional file 1: Tables S2 to S4.

**Table 3:** 1 L treatment patterns, duration and TT2T switch: drug class, administration route and agents

1 L treatment	Patients, n	Median duration of 1 L treatment (95% CI), months^a^	Median TT2T (95% CI), months^a^
All patients	1992	5.9 (5.5, 6.4)	9.1 (8.5, 10.0)
Class			
TK/VEGF inhibitor	1752	6.3 (5.8, 6.7)	9.8 (8.9, 10.4)
mTOR inhibitor	233	3.9 (3.4, 4.8)	6.0 (4.6, 7.1)
Route of administration			
Oral	1674	6.6 (6.0, 7.3)	9.4 (8.6, 10.2)
IV	318	3.4 (2.7, 3.9)	7.3 (6.0, 9.9)
Agent			
Sunitinib (TK; oral)	849	6.5 (5.9, 7.6)	9.4 (8.5, 10.3)
Sorafenib (TK; oral)	62	4.7 (3.3, 9.8)	8.4 (4.8, 12.6)
Pazopanib (TK; oral)	631	7.0 (6.0, 8.0)	9.3 (8.2, 10.6)
Axitinib (TK; oral)	56	12.0 (7.4, 18.8)	14.4 (7.7, 22.3)
Bevacizumab (VEGF; IV)	149	2.8 (1.8, 4.4)	22.5 (11.7, NR)
Everolimus (mTOR; oral)	76	4.0 (3.2, 6.1)	8.0 (3.6, 11.3)
Temsirolimus (mTOR; IV)	157	3.9 (3.0, 4.8)	5.7 (4.3, 6.7)

^a^ Kaplan-Meier methods were used. *NR* not reached

**Fig. 3:**
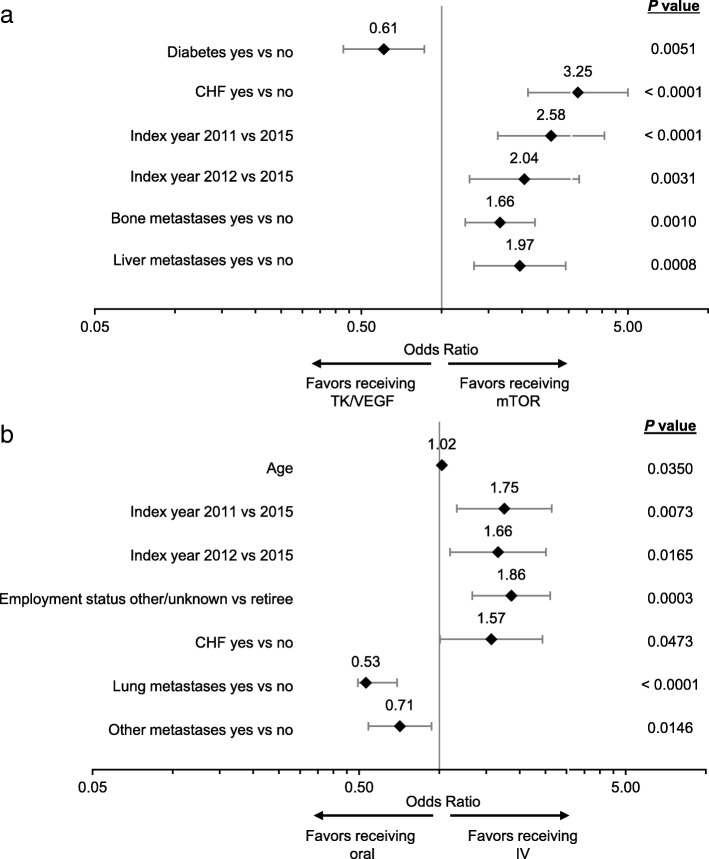
Multivariate analysis. **a** Odds of receiving 1 L mTOR vs TK/VEGF inhibitors. **b** Odds of receiving IV vs orally administered treatment. Only variables found to be significant are plotted. Other variables in the multivariate analysis for the comparison of TK/VEGF inhibitor treatment vs mTOR inhibitor treatment were not significantly different (age, sex, index years 2013 and 2014 vs 2015, DCCI and nephrectomy). Other variables in the multivariate analysis for the comparison of oral vs IV administration that did not reach statistical significance were sex, employment status (active and long-term disability vs retiree), insurance type, DCCI, index years 2013 and 2014 vs 2015 and nephrectomy)

Factors potentially associated with drug class or route of administration were evaluated. Multivariate analyses comparing baseline characteristics of patients treated with TK/VEGF and mTOR inhibitors showed that patients with either baseline congestive heart failure (CHF), secondary bone metastases or secondary liver metastases and those who started 1 L treatment in 2011 or 2012 (vs 2015) were more likely to receive mTOR inhibitors (Fig. 3a). Conversely, patients with baseline diabetes were more likely to receive TK/VEGF-directed agents than those without (Fig. 3a). In multivariate analyses comparing oral vs IV treatments, patients who had baseline secondary lung metastases or other baseline metastases (other than lung/liver/brain/bone) were more likely to receive oral therapy than those who did not. Non-retired patients and those who started 1 L treatment in 2011 or 2012 (vs 2015) were more likely to receive IV than oral treatment (Fig. 3b).

### 1 L treatment duration, TT2T and characterization of 2 L treatments: longitudinal analysis

**Fig. 4:**
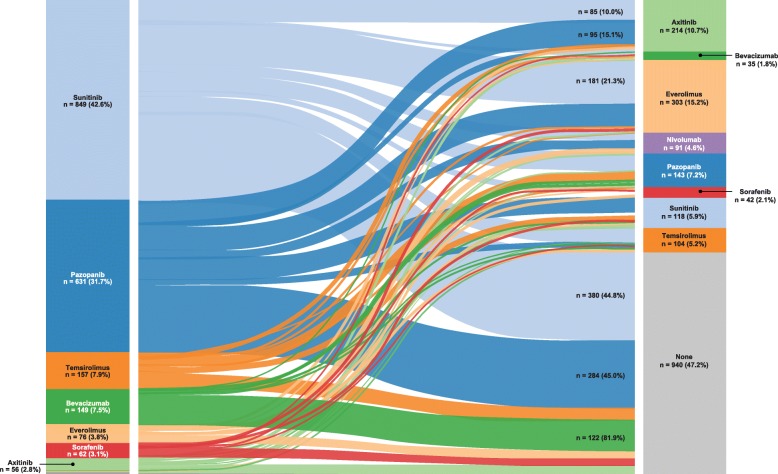
1 L to 2 L treatment flow in patients with mRCC in the Truven Health MarketScan databases. Possible reasons for patients not receiving 2 L treatment include still being on 1 L treatment, no requirement for 2 L treatment, refusal of treatment and death, although these cannot be reliably obtained from the claims database

Among the 1992 patients treated with 1 L targeted therapy, the median duration of 1 L treatment was 5.9 months (Table 3). The median duration of 1 L TK/VEGF-directed (6.3 months) or oral (6.6 months) treatment was longer than that of 1 L mTOR-directed (3.9 months) or IV (3.4 months) treatment. The median TT2T was 9.1 months (Table 3). Among the oral agents, axitinib had the longest median treatment duration (12.0 months) and median TT2T (14.4 months); everolimus had the shortest median treatment duration (4.0 months) and median TT2T (8.0 months). Among the IV agents, the median treatment durations of temsirolimus and bevacizumab were 3.9 and 2.8 months, respectively. The median TT2T was 5.7 months for temsirolimus and 22.5 months for bevacizumab (Table 3). The flow of treatment across lines of therapy is shown in Fig. 4. Of the 1992 patients who received 1 L treatment, 52.8% (*n* = 1052) received a 2 L treatment. The median follow-up durations in patients who did and did not receive 2 L treatment were 17.2 and 14.4 months, respectively. Everolimus was the 2 L drug of choice (15.2% of all 1992 evaluated patients), followed by axitinib (10.7%) and nivolumab (4.6%). Overall, 28% of all patients received 2 L TK/VEGF-directed therapy, and 20% received 2 L mTOR-targeted therapy. Forty-one percent of patients received oral 2 L therapy, and 12% received IV 2 L therapy (including nivolumab).

### IR and time to potentially treatment-related AEs: longitudinal analysis

The three most common, potentially treatment-related AEs occurring during or up to 30 days after 1 L treatment were nausea/vomiting (127.2 per 100 PY), hypertension (69.1 per 100 PY) and renal insufficiency (44.5 per 100 PY) (Table 4). In addition to nausea/vomiting and renal insufficiency, hypertension (in patients who received oral treatment and those treated with TK/VEGF inhibitors) and anemia (in patients who received IV treatment and those treated with mTOR-directed therapy) were among the four most common AEs when evaluated separately by treatment categories.

**Table 4:** AE IRs in patients with mRCC by drug class and administration route

Potentially treatment-related AEs during or < 30 days after 1 L treatment^a^	All patients (*N* = 1992)	TK/VEGF inhibitor (*n* = 1752)	mTOR inhibitor (*n* = 233)	Oral (*n* = 1674)	IV (*n* = 318)
	IR per 100 PY^a^ (Poisson 95% CI)	IR per 100 PY^a^ (Poisson 95% CI)	IR per 100 PY^a^ (Poisson 95% CI)	IR per 100 PY^a^ (Poisson 95% CI)	IR per 100 PY^a^ (Poisson 95% CI)
Fatigue/asthenia	39.8 (36.3, 43.6)	38.2 (34.5, 42.0)	55.2 (41.7, 70.5)	38.7 (34.9, 42.6)	49.3 (37.8, 62.3)
Hypertension	69.1 (61.3, 77.4)	71.7 (63.3, 80.7)	47.8 (29.2, 71.0)	70.2 (61.9, 79.0)	59.2 (37.9, 85.2)
Diarrhea	28.5 (25.6, 31.6)	29.9 (26.7, 33.2)	13.1 (7.3, 20.5)	30.7 (27.5, 34.1)	13.2 8.0, 19.5)
Hand-foot syndrome	4.1 (3.1, 5.2)	4.0 (3.0, 5.2)	3.1 (0.9, 6.9)	4.0 (3.0, 5.2)	4.4 (1.8, 8.3)
Dyspnea	37.6 (34.1, 41.2)	34.7 (31.3, 38.4)	69.6 (53.4, 88.0)	35.9 (32.3, 39.6)	51.7 (39.9, 65.0)
Nausea/vomiting	127.2 (119.0, 135.6)	114.2 (106.3, 122.4)	364.5 (304.6, 429.8)	119.1 (110.8, 127.6)	209.7 (175.6, 246.8)
Back pain	19.9 (17.5, 22.4)	20.3 (17.8, 23.0)	16.2 (9.7, 24.2)	19.8 (17.3, 22.5)	20.8 (14.0, 28.9)
Pain in extremity/limb discomfort	27.8 (24.9, 30.8)	26.6 (23.6, 29.7)	40.9 (29.6, 54.0)	27.1 (24.1, 30.3)	33.0 (24.1, 43.4)
Abdominal pain	31.2 (28.1, 34.5)	30.6 (27.4, 34.0)	35.8 (25.1, 48.4)	32.0 (28.7, 35.6)	25.0 (17.3, 34.1)
Anemia	40.3 (36.6, 44.1)	35.4 (31.8, 39.1)	100.3 (79.1, 123.9)	37.9 (34.1, 41.8)	61.6 (48.0, 76.9)
Hypophosphatemia	1.7 (1.1, 2.5)	1.5 (0.9, 2.2)	2.3 (0.5, 5.6)	1.5 (0.9, 2.3)	3.1 (1.0, 6.4)
Neutropenia	3.9 (3.0, 5.0)	4.2 (3.1,5.3)	1.6 (0.2, 4.3)	4.2 (3.1, 5.4)	1.9 (0.4, 4.5)
Lymphopenia	0.2 (0.0, 0.5)	0.1 (0.0, 0.3)	1.6 (0.2, 4.3)	0.2 (0.0, 0.4)	0.6 (0.0, 2.3)
Hypotension	7.7 (6.3, 9.3)	7.6 (6.2, 9.2)	7.2 (3.3, 12.7)	7.6 (6.1, 9.3)	8.6 (4.6, 13.8)
Proteinuria	5.2 (4.1, 6.5)	5.0 (3.9, 6.3)	7.3 (3.4, 12.9)	4.7 (3.6, 5.9)	9.8 (5.5, 15.4)
Thrombocytopenia	3.0 (2.2, 3.9)	3.0 (2.2, 4.1)	2.3 (0.5, 5.6)	3.1 (2.2, 4.1)	1.9 (0.4, 4.5)
Hepatitis	9.3 (7.7, 11.1)	9.1 (7.5, 11.0)	11.0 (5.7, 18.1)	9.7 (7.9, 11.6)	6.3 (2.9, 11.0)
Thyroid disorders	21.2 (18.6, 24.0)	21.9 (19.1, 24.9)	14.9 (8.5, 23.0)	22.9 (20.0, 26.0)	9.5 (5.0, 15.3)
Renal insufficiency	44.5 (38.9, 50.5)	42.3 (36.6, 48.4)	60.8 (38.9, 87.4)	43.0 (37.2, 49.3)	58.3 (39.0, 81.3)
Adrenal insufficiency	6.3 (5.0, 7.7)	5.8 (4.5, 7.2)	10.4 (5.4, 17.0)	5.9 (4.6, 7.4)	9.1 (4.8, 14.6)
Pneumonitis	27.3 (24.4, 30.2)	24.6 (21.8, 27.6)	56.2 (42.4, 71.8)	25.2 (22.4, 28.3)	43.6 (33.3, 55.3)
Colitis	0.5 (0.2, 0.9)	0.5 (0.2, 0.9)	0.8 (0.0, 2.9)	0.4 (0.1, 0.8)	1.3 (0.2, 3.5)
Guillain-Barré syndrome	0.1 (0.0, 0.3)	0.1 (0.0, 0.3)	0	0.1 (0.0, 0.3)	0
Meningoencephalitis	0.5 (0.2, 0.9)	0.5 (0.2, 1.0)	0	0.5 (0.2, 0.9)	0.6 (0.0, 2.3)
Myasthenia gravis	0	0	0	0	0
Rash	10.1 (8.5, 11.9)	9.5 (7.9, 11.3)	14.3 (8.4, 21.9)	9.9 (8.2, 11.7)	11.9 (7.1, 18.0)

^a^ Washout period of 30 days prior to the index date was applied when identifying incidence events for all AEs, except for chronic conditions, where a 365-day washout period was applied. If a patient did not have an event, their person-time was counted up to 30 days after the end of first-line treatment or the end of eligibility, whichever occurred first

Based on time between the claims indicating the initiation of a drug and the initial AE that may have required medical attention, substantial latency of onset (median time to first AE claim) was observed for several potentially treatment-related toxicities in patients treated with 1 L TK/VEGF-directed treatment, including nausea/vomiting (21 days), fatigue/asthenia (72 days), thyroid disorders (133 days), abdominal pain (99 days), diarrhea (108 days), pneumonitis (83 days), hepatitis (154 days), adrenal insufficiency (119 days), neutropenia (110 days) and colitis (97 days) (Table 5).

**Table 5:** Time to onset of AEs by drug class and route of administration

Adverse event	All patients (*N* = 1992)	TK/VEGF inhibitor (*n* = 1752)	mTOR inhibitor (*n* = 233)	Oral (*n* = 1674)	IV (*n* = 318)
	Median, days (Q1–Q3)	Median, days (Q1–Q3)	Median, days (Q1–Q3)	Median, days (Q1–Q3)	Median, days (Q1–Q3)
Fatigue/asthenia	72.0 (29.0, 157.0)	74.0 (28.0, 168.0)	63.0 (35.5, 119.0)	74.0 (28.0, 165.0)	58.5 (29.0, 118.0)
Hypertension	72.0 (28.0, 156.0)	70.0 (28.0, 151.0)	116.5 (42.0, 240.5)	72.0 (29.0, 163.0)	76.5 (11.5, 138.5)
Diarrhea	108.0 (45.0, 211.0)	111.0 (48.5, 215.5)	46.0 (25.0, 194.0)	110.0 (48.0, 214.0)	46.5 (21.5, 194.5)
Hand-foot syndrome	93.5 (43.0, 218.0)	109.0 (54.0, 231.5.0)	30.0 (20.5, 53.0)	112.0 (57.0, 241.0)	30.0 (10.0, 33.0)
Dyspnea	88.0 (28.0, 210.0)	94.0 (29.0, 227.0)	68.5 (23.0, 156.0)	95.0 (31.0, 226.0)	52.0 (20.0, 146.0)
Nausea/vomiting	21.0 (4.0, 78.0)	27.0 (7.0, 89.0)	0	28.0 (8.0, 90.0)	0
Back pain	90.0 (31.0, 198.0)	90.5 (34.0, 197.50)	89.0 (22.0, 287.0)	93.0 (34.0, 198.0)	75.0 (20.0, 196.0)
Pain in extremity/limb discomfort	112.0 (46.0, 238.0)	113.0 (44.0, 238.0)	95.0 (48.0, 240.0)	113.0 (46.5, 246.0)	83.0 (44.0, 165.0)
Abdominal pain	98.5 (35.0, 226.5)	104.5 (36.0, 229.0)	87.0 (32.5, 195.0)	104.5 (37.0, 229.0)	63.5 (30.0, 176.0)
Anemia	85.0 (32.5, 183.5)	93.0 (35.0, 200.0)	66.0 (26.0, 132.0)	93.5 (35.0, 201.0)	64.5 (18.0, 118.0)
Hypophosphatemia	100.0 (56.0, 138.0)	102.0 (60.0, 136.5)	299.0 (43.0, 498.0)	110.0 (60.0, 146.0)	43.0 (9.0, 104.0)
Neutropenia	110.0 (47.5, 242.5)	117.0 (48.0, 245.0)	37.5 (19.0, 56.0)	115.0 (48.0, 240.0)	56.0 (19.0, 465.0)
Lymphopenia	108.0 (95.0, 136.0)	95.0 (95.0, 95.0)	122.0 (108.0, 136.0)	115.5 (95.0, 136.0)	108.0 (108.0, 108.0)
Hypotension	105.0 (42.0, 222.0)	117.5 (44.0, 239.0)	58.0 (51.0, 179.0)	111.0 (43.0, 230.0)	56.0 (41.0, 191.0)
Proteinuria	80.5 (29.0, 245.0)	84.0 (30.0, 235.0)	56.0 (26.0, 257.0)	108.0 (41.0, 336.0)	30.0 (20.0, 84.0)
Thrombocytopenia	97.0 (50.0, 188.0)	93.5 (48.5, 194.0)	112.0 (52.0, 140.0)	87.0 (48.5, 187.0)	140.0 (112.0, 256.0)
Hepatitis	154.0 (71.0, 256.0)	148.0 (71.0, 253.0)	209.0 (128.0, 326.0)	150.5 (71.0, 256.0)	190.0 (166.0, 234.0)
Thyroid disorders	133.0 (75.0, 256.0)	134.0 (79.0, 259.0)	91.0 (55.0, 196.0)	133.5 (79.0, 259.0)	131.0 (62.0, 175.0)
Renal insufficiency	94.0 (33.0, 217.0)	95.0 (39.5, 225.5)	90.5 (18.0, 62.5)	97.0 (41.0, 231.0)	61.0 (14.0, 133.0)
Adrenal insufficiency	119.0 (69.0, 213.0)	108.0 (67.0, 227.0)	147.0 (118.0, 200.0)	110.0 (70.0, 218.0)	132.0 (4.0, 201.0)
Pneumonitis	83.0 (29.0, 209.0)	87.0 (31.0, 223.0)	73.5 (21.5, 155.5)	90.0 (31.0, 223.0)	68.5 (19.5, 150.5)
Colitis	97.0 (43.0, 139.0)	101.5 (66.0, 139.0)	0	106.0 (97.0, 139.0)	21.5 (0.0, 43.0)
Guillain-Barré syndrome	52.0 (52.0, 52.0)	52.0 (52.0, 52.0)	0	52.0 (52.0, 52.0)	0
Meningoencephalitis	122.0 (75.0, 159.0)	122.0 (75.0, 159.0)	0	112.0 (75.0, 159.0)	122.0 (122.0, 122.0
Myasthenia gravis	0	0	0	0	0
Rash	79.0 (37.5, 214.0)	83.0 (44.0, 220.0)	55.0 (23.0, 149.0)	82.5 (47.0, 213.0)	28.0 (10.0, 224.0)

## Discussion

As the number of 1 L and 2 L treatment options for mRCC increases, and the role of effective sequencing of agents evolves, a thorough understanding of the treatment patterns and AE profiles of each drug class in the real-world setting will be critical to providing medical benchmarks, assessing adherence to guidelines and informing benefit:risk decisions in selecting the appropriate mRCC treatment. This retrospective, claims-based analysis evaluated treatment patterns and AEs among a large, real-world, US population of patients with mRCC. A cross-sectional analysis was performed to better understand the patterns of 1 L treatment initiation over a 10-year period, and a longitudinal analysis provided information of treatment choice, baseline correlates and associated AEs in patients treated in routine medical practice.

The cross-sectional trend analysis demonstrated that 1 L treatment initiation patterns for mRCC generally reflected the US Food and Drug Administration approvals and NCCN treatment guidelines, with a few exceptions. The rapid uptake of the TK-targeting agents sunitinib (approved in 2006) and pazopanib (approved in 2009) was noted, establishing TK/VEGF-directed treatment as the most widely prescribed drug class initiated during the study period. Throughout the study period, oral treatments were also more commonly used than IV treatments. These patterns of TK/VEGF inhibitors and oral agents were also recapitulated in the longitudinal analysis. These findings are consistent with those from studies detailing the widespread use of TK inhibitors [26], and the less frequent use of sorafenib and axitinib in the 1 L setting is also consistent with NCCN recommendations for these TK inhibitors [8]. Of note was the finding that the use of bevacizumab as monotherapy was more prevalent than as combination therapy with IFN-α. These findings suggest that increased costs and IFN-related toxicities may render bevacizumab more attractive as monotherapy than as combination therapy with IFN [27]. Moreover, Phase II data support the use of bevacizumab as 1 L treatment and salvage therapy [28, 29]. Additionally, a considerable number of patients received everolimus in the 1 L setting despite the lack of indication in this setting, which reflects the wide variability seen in provider preference and clinical experience.

Provider preferences, patient history and known toxicities associated with drug classes may drive 1 L treatment choice, although it is difficult to ascertain from claims data which characteristics influenced the specific choices of mRCC treatment. In this study, CHF and a DCCI score ≥ 4 were independent predictors of IV treatment choice, while patients with lung metastases were less likely to receive IV treatment than were those without lung metastases. Patients with diabetes were less likely to receive mTOR-directed therapy, while patients with comorbid CHF, liver metastases or bone metastases were more likely to receive mTOR-directed therapy. These data are in agreement with the known cardiotoxicity associated with both sunitinib and pazopanib [23, 25], such as cardiac dysfunction (sunitinib, 11% frequency; pazopanib, 13%) and myocardial infarction/ischemia (sunitinib, 4% frequency; pazopanib, 2%) [11]. Similarly, a preference for TK/VEGF inhibitors in those with diabetes can be explained by the fact that hyperglycemia is a well-known AE associated with mTOR inhibitors (observed in 26% of patients treated with temsirolimus [30] and 50% treated with everolimus [31]) .

Duration of treatment also varies by agent and may influence treatment choice. Median duration of TK/VEGF-directed treatment was much longer than that of mTOR-directed treatment (6.3 and 3.9 months); similarly, oral treatment duration was nearly twice that of IV treatment (6.6 and 3.4 months). The median durations of sunitinib and pazopanib treatment were similar to each other (6.5 and 7.0 months, respectively), but were longer than those of sorafenib (4.7 months), everolimus (4.0 months), and temsirolimus (3.9 months). Reported results of real-world studies are mixed. For example, the observed sunitinib and sorafenib treatment durations were similar to those reported by Feinberg et al. (5.9 and 5.5 months, respectively) [32] and only slightly longer than those reported by Miller et al. (5.6 and 5.3 months, respectively), who also reported durations of 5.3 months for pazopanib and 4.5 months for everolimus [14]. However, these durations are slightly longer than those reported by Hess et al. (3.2 and 4.0 months, respectively; 2.6 months for temsirolimus) [33] and Vogelzang et al. (sunitinib, 4.1 months; pazopanib 4.8 months) [15]. However, it should be noted that direct cross-study comparisons are not possible based on different analysis methods and populations. Treatment durations reported in the randomized clinical trial setting are also sometimes mixed and are not dissimilar to the results from this study. For instance, Phase III data have shown 1 L median treatment durations of 7.6 and 8.0 months, respectively, for TK inhibitors sunitinib and pazopanib [11] and 3.9 months for the mTOR inhibitor temsirolimus [30]. Collectively, these data suggest that there are potential differences in patient selection, outcomes measurement and patient preference between real-world data studies and randomized clinical trials that could have implications for future clinical trial design. These differences may reflect the difficulty in (1) maintaining adequate dosing with these relatively toxic agents and (2) reproducing clinical trial results in the real-world setting for mRCC.

Potential differences in toxicities between the 1 L treatments in this study vs those previously seen were also evaluated, and it was noted that the most common AEs associated with each drug class were generally consistent with previously reported results [34–37] and with the product labels [19–25]. Notably, however, substantial latency of onset was observed for several potentially treatment-related toxicities in patients treated with both TK/VEGF- and mTOR-inhibitor classes, which was much different in clinical practice (i.e., onset of fatigue, hypertension and hepatic dysfunction generally occurs quickly). AE latency in the database may be due to capture of only the toxicities that generate a medical claim and may suggest that the toxicities that are observed early on may not receive medical care in clinical practice. There were also AEs potentially associated with checkpoint inhibitors. The latency of these AEs was similar to that observed with checkpoint inhibitors [38, 39]. These results suggest that attributing toxicities to TK/VEGF-directed therapies vs checkpoint inhibitors, when used in combination, may be challenging due to overlapping toxicities.

In contrast to the 1 L treatment data, 2 L treatment data were less defined. In this study, nearly half of all patients did not have evidence of receiving 2 L treatment (47% [*n* = 940]); however, there is no reliable way to determine the reasons for this from a claims database. Patients who did not receive 2 L therapy in this study may either still be receiving 1 L targeted therapy (and were not captured in the study due to the follow-up period ending) or have attained sustained remission, died before receiving 2 L therapy, refused 2 L treatment or did not receive 2 L therapy for unknown reasons. Consistent with other studies, most patients treated with a 1 L oral TK inhibitor who received 2 L therapy were switched to 2 L mTOR-directed treatment, primarily everolimus [26]; 2 L treatment choice warrants further investigation.

This study had several strengths and limitations. Strengths include the large number of 1 L–treated patients with mRCC over time, including those treated with TK/VEGF- or mTOR-directed therapy. The use of the MarketScan databases also provided strength to this study due to the inclusion of the full continuum of care in all inpatient and outpatient settings, as well as retail and specialty pharmacies, and the longitudinal tracking of patient information. However, the MarketScan databases also have limitations, as they are inherently restricted to insured patients and provide limited social background and demographic data. Secondary metastasis codes were not required for identification of patients with mRCC since the 1 L treatments are approved in the metastatic setting only and to avoid exclusion of eligible patients due to potential underreporting of secondary metastasis codes. However, the study could have included patients who received the treatments off-label in the adjuvant/neoadjuvant setting. Further, administrative claims-based data are prone to coding and data entry errors. Potential underreporting of secondary metastasis and baseline comorbidities may have affected the point estimates in the multivariate models, and the distribution of these variables may not be representative of the RCC population in clinical practice. Moreover, only AEs of sufficient severity to prompt medical attention and generate a claim could be identified, which may have led to underreported IRs. It was also not possible to discern from the claims data whether an AE was reported due to a drug reaction, RCC progression or other cause, nor was it possible to know the severity/grade of the AE. Further, it is unknown from the claims data whether 2 L treatment was received due to toxicity or disease progression. In this study, the treatment duration end date was calculated by adding the days’ supply of oral treatment and cycle length for IV treatment. Since most patients receive their oral drug supply 1 to 3 months in advance, the treatment duration for oral medications may have been overestimated. Additionally, the proportion of patients who switched treatment may have been underreported due to the limited follow-up in this study (median of ≈ 16 months after the index date). However, this median follow-up may be of limited concern considering the low survival rates in this patient population [40, 41]. Lastly, recent approvals (e.g., cabozantinib and nivolumab ± ipilimumab) and treatment recommendations were not captured given that these agents were not approved during the period covered by the analysis [42–44].

## Conclusions

In this retrospective, claims-based study of patients with mRCC in the Truven Health MarketScan databases, 10-year prescribing trends showed the transition from cytokine-based treatment to targeted agents and a similar shift from IV to oral agents. Observed 1 L treatment patterns generally suggested good adherence to NCCN guideline recommendations, although treatment durations across the literature appear variable. In all patients and those treated with TK/VEGF- and mTOR-directed 1 L treatment, the three most common AEs were hypertension, nausea/vomiting and renal insufficiency. The 2 L treatments included a wide variety of agents for mRCC. With new agents and combinations (e.g., checkpoint inhibitor therapy) emerging following this study period (through 2016), these data will be useful in providing medical benchmarks for contemporary mRCC therapy.

## Additional file

Additional file 1: **Table S1.** Listing of AEs included for analysis, with ICD-9/ICD-10-CM codes. **Table S2.** Baseline characteristics by TK inhibitor type. **Table S3.** Baseline characteristics by VEGF inhibitor type. **Table S4.** Baseline characteristics by mTOR inhibitor type. (DOCX 71 kb)

## Abbreviations

1 L: First-line
2 L: Second-line
AE: Adverse event
CHF: Congestive heart failure
DCCI: Deyo-Charlson Comorbidity Index
IFN: Interferon
IL: Interleukin
IR: Incidence rate
IV: Intravenous
mRCC: Metastatic renal cell carcinoma
mTOR: Mechanistic target of rapamycin kinase
NCCN: National Comprehensive Cancer Network
ORR: Objective response rate
OS: Overall survival
PFS: Progression-free survival
PY: Patient-years
Q: Quartile
RCC: Renal cell carcinoma
TK: Tyrosine kinase
TT2T: Time to 2 L treatment
VEGF: Vascular endothelial growth factor
