# Supplementary material for: Real-world treatment patterns and adverse events in metastatic renal cell carcinoma from a large US claims database
Source: BMC Cancer. 2019 Jun 7;19:548. doi: 10.1186/s12885-019-5716-z (PMC6555983; doi:10.1186/s12885-019-5716-z)
Supplement: Supplementary file 1 — Table S1. Listing of AEs included for analysis, with ICD-9/ICD-10-CM codes. Table S2. Baseline characteristics by TK inhibitor type. Table S3. Baseline characteristics by VEGF inhibitor type. Table S4. Baseline characteristics by mTOR inhibitor type. (DOCX 71 kb) [file 12885_2019_5716_MOESM1_ESM.docx]

Additional File 1. SUPPLEMENTARY TABLES

**Table S1. Listing of AEs included for analysis, with *ICD-9/ICD-10-CM* codes**

| **Grade 3-4 AEs (occurring in ≥ 5% of patients)** | ***ICD-9/ICD-10-CM* codes** |
| --- | --- |
| Fatigue/asthenia | 780.7, 799.3, 780.71, 780.72, R53.2, R53.8, R53.81, R53.82 |
| Hypertension | 362.11, 401.x, 402.x, 403.x, 404.x, 405.x, 437.2x, 642.0x, 642.1x, 642.2x, H35.03, H35.031, H35.032, H35.033, H35.039, I10, I11, I11.0, I11.9, I12, I12.0, I12.9, I13, I13.0, I13.1, I131.0, I131.1, I13.2, I15, I15.0, I15.1, I15.2, I15.8, I15.9, I67.4, O10.x |
| Diarrhea | 564.5, 787.91, K59.1, R19.7 or use of agents (THERCLS:148) |
| Hand-foot syndrome | 693, L27 |
| Dyspnea | 518.81, 518.82, 786.0x, R06.0, R06.00, R06.01, R06.02, R06.09, R06.2,  R06.3, R06.4, R06.8, R06.81, R06.82, R06.89, R06.9 |
| Nausea/vomiting | 536.2x, 578.0x, 787.0x, R11, K92.0 or use of antiemetics  (Appendix A – antiemetics) (THERCLS: 160) |
| Back pain | 724.1, 724.2, 724.5, M54.6, M54.5, M54.89, M54.9 |
| Pain in extremity/limb discomfort | 729.5, M79.6x |
| Abdominal pain | 789, R10.1, R10.10, R10.11, R10.12, R10.13, R10.3, R10.30, R10.31, R10.32, R10.33, R10.84, R10.9 |
| Anemia | 284.xx, 285.xx, D61.0, D61.01, D61.09, D61.8, D61.81, D61.810, D61.811, D61.818, D61.82, D61.89, D61.9, D63.0, D64, D64.0, D64.1, D64.2, D64.3, D64.4, D64.8, D64.81, D64.89, D64.9 |
| Hypophosphatemia | 275.3, E83.3, E83.30, E83.31, E83.32, E83.39 |
| Neutropenia | 288.0, 288.03, D70.9, D70.1, D70.2 |
| Lymphopenia | 288.51, D72.810 |
| Hypotension | 458.x, I95.x |
| Proteinuria | 791.0, R80.x |
| Thrombocytopenia | 287.4x, D69.51, D69.59 |
| Hepatitis | 571.42, 573.3, 573.8, K75.4, K71.6, K75.9, K76.89 |
| Thyroid disorders | 240.x, 241.x, 242.x, 243.x, 244.x, 245.x, E00, E00.0, E00.1, E00.2, E00.9, E04, E04.0, E04.1, E04.2, E04.8, E04.9, E05, E05.0, E05.00, E05.01, E05.1, E05.10, E05.11, E05.2, E05.20, E05.21, E05.3, E05.30, E05.31, E05.4, E05.40, E05.41, E05.8, E05.80, E05.81, E05.9, E05.90, E05.91, E06, E06.0, E06.1, E06.2, E06.3, E06.4, E06.5, E06.9, E89.0 |
| Renal disorders | 593.9, 584, 585, 586, N17-N19, N28.9 |
| Adrenal insufficiency | 255.xx, E24-E27 |
| Pneumonitis | 786.05, R06.02 |
| Colitis | 556.x, K51.x |
| Guillain-Barré syndrome | 357.0, G61.0 |
| Meningoencephalitis | 320.x, 321.x, 322.x, 323.x, 324.0, 325.x, B45.1, G00, G00.0, G00.1, G00.2, G00.3, G00.8, G00.9, G02, G03.0, G03.1, G03.8, G03.9, G04.01, G04.02, G05, G05.3, G05.4, G92, G04.90, G04.91, G04.2, G08 |
| Myasthenia gravis | 358.x, G70.x |
| Rash | 693.0, 708.8, 708.9, 782.1, L27.0, L27.1, L50.9, L50.6, R21 |

CM, Clinical Modification; *ICD, International Classification of Diseases*.

Table S2. Baseline characteristics by TK inhibitor type

| **Characteristic** | **Sunitinib**  **(n = 849)** | | **Sorafenib**  **(n = 62)** | | | **Pazopanib**  **(n = 631)** | | **Axitinib**  **(n = 56)** | |
| --- | --- | --- | --- | --- | --- | --- | --- | --- | --- |
|  | **n** | **%** | **n** | **%** | **n** | | **%** | **n** | **%** |
| **Age at diagnosis, median  (Q1-Q3), years** | 61  (56-68) | | 61  (58-71) | | | 61  (56-70) | | 59  (55-64) | |
| **Male** | 600 | 70.7 | 48 | 77.4 | 438 | | 69.4 | 36 | 64.3 |
| **Employment status** | | | | | | | | | |
| Active | 280 | 33.0 | 21 | 33.9 | 213 | | 33.8 | 26 | 46.4 |
| Retiree | 310 | 36.5 | 23 | 37.1 | 246 | | 39.0 | 12 | 21.4 |
| Long-term disability | 4 | 0.5 | 1 | 1.6 | 2 | | 0.3 | 1 | 1.8 |
| Other/unknown | 255 | 30.0 | 17 | 27.4 | 170 | | 26.9 | 17 | 30.4 |
| **Region** | | | | | | | | | |
| Northeast | 152 | 17.9 | 9 | 14.5 | 115 | | 18.2 | 14 | 25.0 |
| North Central | 229 | 27.0 | 13 | 21.0 | 179 | | 28.4 | 18 | 32.1 |
| South | 300 | 35.3 | 26 | 41.9 | 241 | | 38.2 | 19 | 33.9 |
| West | 159 | 18.7 | 14 | 22.6 | 89 | | 14.1 | 4 | 7.1 |
| Unknown^a^ | 9 | 1.1 | 0 | 0 | 7 | | 1.1 | 1 | 1.8 |
| **Metropolitan statistical area** | | | | | | | | | |
| Urban | 674 | 79.4 | 57 | 91.9 | 511 | | 81.0 | 47 | 83.9 |
| Rural | 175 | 20.6 | 5 | 8.1 | 120 | | 19.0 | 9 | 16.1 |
| **Insurance plan type** | | | | | | | | | |
| Comprehensive | 143 | 16.8 | 13 | 21.0 | 112 | | 17.8 | 8 | 14.3 |
| HMO | 99 | 11.7 | 14 | 22.6 | 64 | | 10.1 | 6 | 10.7 |
| POS | 58 | 6.8 | 2 | 3.2 | 51 | | 8.1 | 2 | 3.6 |
| PPO | 447 | 52.7 | 30 | 48.4 | 343 | | 54.4 | 32 | 57.1 |
| Other | 102 | 12.0 | 3 | 4.8 | 61 | | 9.7 | 8 | 14.3 |
| **Insurance type** | | | | | | | | | |
| Commercial | 576 | 67.8 | 39 | 62.9 | 396 | | 62.8 | 45 | 80.4 |
| Medicare | 273 | 32.2 | 23 | 37.1 | 235 | | 37.3 | 11 | 19.6 |
| **Index year** | | | | | | | | | |
| 2011 | 226 | 26.6 | 24 | 38.7 | 71 | | 11.3 | 0 | 0 |
| 2012 | 181 | 21.3 | 11 | 17.7 | 113 | | 17.9 | 11 | 19.6 |
| 2013 | 188 | 22.1 | 14 | 22.6 | 149 | | 23.6 | 11 | 19.6 |
| 2014 | 126 | 14.8 | 7 | 11.3 | 153 | | 24.2 | 11 | 19.6 |
| 2015 | 128 | 15.1 | 6 | 9.7 | 145 | | 23.0 | 23 | 41.1 |
| **Mean DCCI score  (Q1, Q3)** | 0.64 (0, 1) | | 1.11 (0, 2) | | 0.63 (0, 1) | | | 0.63 (0, 1) | |
| **Comorbidities** | | | | | | | | | |
| Diabetes | 231 | 27.2 | 22 | 35.5 | 164 | | 26.0 | 14 | 25.0 |
| CKD | 145 | 17.1 | 17 | 27.4 | 128 | | 20.3 | 11 | 19.6 |
| Liver disease | 145 | 17.1 | 19 | 30.7 | 110 | | 17.4 | 14 | 25.0 |
| COPD | 115 | 13.6 | 12 | 19.4 | 69 | | 10.9 | 5 | 8.9 |
| CHF | 53 | 6.2 | 8 | 12.9 | 33 | | 5.2 | 4 | 7.1 |

DCCI, Deyo-Charlson Comorbidity Index; CHF, congestive heart failure; CKD, chronic kidney disease; COPD, chronic obstructive pulmonary disease; HMO, health maintenance organization; POS, point-of-service; PPO, preferred provider organization; Q, quartile; TK, tyrosine kinase.

**Table S3. Baseline characteristics by VEGF inhibitor type**

| **Characteristic** | **Bevacizumab**  **(n = 149)** | | **Bevacizumab + IFN-α**  **(n = 5)** | |
| --- | --- | --- | --- | --- |
|  | **n** | **%** | **n** | **%** |
| **Age at diagnosis, median (Q1-Q3), years** | 69 (58-80) | | 66 (65-73) | |
| **Male** | 74 | 63.8 | 1 | 20.0 |
| **Employment status** | | | | |
| Active | 28 | 18.8 | 0 | 0 |
| Retiree | 58 | 38.9 | 3 | 60.0 |
| Long-term disability | 3 | 2.0 | 0 | 0 |
| Other/unknown | 60 | 40.3 | 2 | 40.0 |
| **Region** | | | | |
| Northeast | 37 | 24.8 | 0 | 0 |
| North Central | 26 | 17.5 | 2 | 40.0 |
| South | 58 | 38.9 | 1 | 20.0 |
| West | 28 | 18.8 | 2 | 40.0 |
| Unknown^a^ | 0 | 0 | 0 | 0 |
| **Metropolitan statistical area** | | | | |
| Urban | 131 | 87.9 | 4 | 80.0 |
| Rural | 18 | 12.1 | 1 | 20.0 |
| **Insurance plan type** | | | | |
| Comprehensive | 35 | 23.5 | 2 | 40.0 |
| HMO | 21 | 14.1 | 0 | 0 |
| POS | 17 | 11.4 | 0 | 0 |
| PPO | 71 | 47.7 | 3 | 60.0 |
| Other | 5 | 3.4 | 0 | 0 |
| **Insurance type** | | | | |
| Commercial | 63 | 42.3 | 1 | 20.0 |
| Medicare | 86 | 57.7 | 4 | 80.0 |
| **Index year** | | | | |
| 2011 | 28 | 18.8 | 1 | 20.0 |
| 2012 | 31 | 20.8 | 3 | 60.0 |
| 2013 | 29 | 19.5 | 1 | 20.0 |
| 2014 | 30 | 20.1 | 0 | 0 |
| 2015 | 31 | 20.8 | 0 | 0 |
| **Mean DCCI score (Q1, Q3)** | 1.71 (0, 2) | | 0.20 (0, 0) | |
| **Comorbidities** | | | | |
| Diabetes | 52 | 34.9 | 1 | 20.0 |
| CKD | 45 | 30.2 | 0 | 0 |
| Liver disease | 17 | 11.4 | 2 | 40.0 |
| COPD | 16 | 10.7 | 0 | 0 |
| CHF | 15 | 10.1 | 0 | 0 |

DCCI, Deyo-Charlson Comorbidity Index; CHF, congestive heart failure; CKD, chronic kidney disease; COPD, chronic obstructive pulmonary disease; HMO, health maintenance organization; POS, point-of-service; PPO, preferred provider organization; Q, quartile; VEGF, vascular endothelial growth factor.

**Table S4. Baseline characteristics by mTOR inhibitor type**

| **Characteristic** | **Temsirolimus**  **(n = 157)** | | **Everolimus**  **(n = 76)** | |
| --- | --- | --- | --- | --- |
|  | **n** | **%** | **n** | **%** |
| **Age at diagnosis, median (Q1-Q3), years** | 62 (57-71) | | 63 (56-71) | |
| **Male** | 112 | 71.3 | 54 | 71.1 |
| **Employment status** | | | | |
| Active | 46 | 29.3 | 23 | 30.3 |
| Retiree | 52 | 33.1 | 26 | 34.2 |
| Long-term disability | 1 | 0.6 | 0 | 0 |
| Other/unknown | 58 | 36.9 | 27 | 35.5 |
| **Region** | | | | |
| Northeast | 20 | 12.7 | 13 | 17.1 |
| North Central | 40 | 25.5 | 12 | 15.8 |
| South | 68 | 43.3 | 36 | 47.4 |
| West | 26 | 16.6 | 14 | 18.4 |
| Unknown^a^ | 3 | 1.9 | 1 | 1.3 |
| **Metropolitan statistical area** | | | | |
| Urban | 125 | 79.6 | 70 | 92.1 |
| Rural | 32 | 20.4 | 6 | 7.8 |
| **Insurance plan type** | | | | |
| Comprehensive | 33 | 21.0 | 12 | 15.8 |
| HMO | 16 | 10.2 | 6 | 7.9 |
| POS | 10 | 6.4 | 7 | 9.2 |
| PPO | 81 | 51.6 | 44 | 57.9 |
| Other | 17 | 10.8 | 7 | 9.2 |
| **Insurance type** | | | | |
| Commercial | 98 | 62.4 | 44 | 57.9 |
| Medicare | 59 | 37.6 | 32 | 42.1 |
| **Index year** | | | | |
| 2011 | 58 | 36.9 | 20 | 26.3 |
| 2012 | 45 | 28.7 | 17 | 22.4 |
| 2013 | 14 | 8.9 | 11 | 14.5 |
| 2014 | 26 | 16.6 | 12 | 15.8 |
| 2015 | 14 | 8.9 | 16 | 21.1 |
| **Mean DCCI score (Q1, Q3)** | 0.75 (0, 1) | | 0.79 (0, 1) | |
| **Comorbidities** | | | | |
| Diabetes | 37 | 23.6 | 11 | 14.5 |
| CKD | 38 | 24.2 | 16 | 21.1 |
| Liver disease | 22 | 14.0 | 18 | 23.7 |
| COPD | 22 | 14.0 | 11 | 14.5 |
| CHF | 24 | 15.3 | 13 | 17.1 |

DCCI, Deyo-Charlson Comorbidity Index; CHF, congestive heart failure; CKD, chronic kidney disease; COPD, chronic obstructive pulmonary disease; HMO, health maintenance organization; mTOR, mechanistic target of rapamycin; POS, point-of-service; PPO, preferred provider organization; Q, quartile.
